# Essential Health Services Delivery Status During COVID-19 Pandemic in Ethiopia: A National Mixed-Methods Survey of Primary Healthcare Units

**DOI:** 10.4314/ejhs.v33i2.2S

**Published:** 2023-10

**Authors:** Elias Ali Yesuf, Biru Abdisa, Habtamu Sime, Enku Kifle Alemu, Netsanet Abera Asseffa, Meskerem Jisso, Alemu Tamiso, Akalewold Alemayehu, Rekiku Fikre, Abdurezak Umer, Mesfin Kebede, Hussen Mohammed, Bekele Yazie, Kassu Ketema Gurmu, Kassahun Dessie Gashu, Dessies Abebaw Angaw, Berhanu Fikadie Endehabtu, Binyam Tilahun, Tajebew Zayede Gonete

**Affiliations:** 1 Jimma University, Institute of Health, Ethiopia; 2 Hawassa University, College of Medicine and Health Sciences, Ethiopia; 3 Dire Dawa University, College of Medicine and Health Sciences, Ethiopia; 4 World Health Organization Country Office for Ethiopia, Universal Health Coverage/Life Course, Health System Strengthening Team, Addis Ababa, Ethiopia; 5 University of Gonder, College of Medicine and Health Science, Institute of Public Health, Ethiopia

**Keywords:** COVID-19, essential health services, primary healthcare, Ethiopia

## Abstract

**Background:**

Essential health services are a package of services critical to improve health outcomes. COVID-19 pandemic disrupts essential health services. However, the level of essential health service disruption due to COVID-19 in Ethiopia is not clear. This study aimed at measuring the status of delivery of essential health services in Ethiopia during COVID-19.

**Methods:**

A national mixed-methods cross-sectional survey was conducted. It was undertaken in Amhara (10 districts), Oromia (eight districts), Sidama (six districts), Southern Nations, Nationalities, and People's Region (16 districts), and Dire Dawa City Administration. A total of 452 health facilities were surveyed. Data were collected using face-to-face interview. Descriptive analysis was undertaken. Qualitative data was analyzed thematically.

**Results:**

The woredas (districts) and health facilities which adopted essential health services before the COVID-19 pandemic were 81.4% and 51.2%, respectively. Nearly all health centers provided antenatal care services. Blood pressure measuring apparatus and delivery set were available in all health centers. However, only 50% of health centers had radiant warmer. Malnutrition services were provided by 47% of rural health centers. Moreover, a functional incinerator was available in only 41% of health centers. The provision of cardiovascular disease management was at 27.2%. Furthermore, HIV/AIDS treatment was provided by 43.5% of health facilities.

**Conclusion:**

The adoption of lists of essential health services was optimal. The status of delivery of essential health services was high for maternal healthcare. Neonatal care at birth, malnutrition treatment, and cardiovascular disease management were low. The district health system should strive more to maintain essential health services.

## Introduction

An organized and prepared health system has the capacity to maintain access to high quality essential health services (1,2) Reproductive, Maternal, Newborn, and Child Health (RMNCH) refers to health challenges and interventions that affect women before, during, and after pregnancy, as well as newborns, infants, and children (3). It is critical to provide maternal and child health services to ensure positive outcomes. Health systems of Low- and Middle-Income countries are vulnerable to pandemic caseloads, such as COVID-19 (4).

Sexual and reproductive health and rights, specifically obstetric services were the center of both global and national agenda. In Ethiopia, reproductive health with a greater focus on obstetric care has been on the agenda for the last two decades (5). A policy analysis revealed that even though proper attention was given to family planning and maternal health, programs were not prepared for female genital mutilation. Moreover, infertility treatment and gynecologic tumors were not exempted (6).

The main reproductive health problems in Ethiopia are preventable. There was improvement in sexual and reproductive health in Ethiopia in the past two decades (7) particularly utilization of family planning services increased fivefold. Together with safe abortion services it led to a decline in Maternal Mortality Ratio (8).

Family planning has important roles in the lives of individuals, communities and populations (9). Over the last few decades, Ethiopia has made significant progress towards the achievement of universal health care, notably in terms of access to and utilization of family planning services. On the other hand, family planning services have been interrupted by the COVID-19 pandemic. For example, in Kenya, d14 million women were unable to use contraception because of COVID-19 (10).

According to the World Health Organization, Non-Communicable Diseases (NCDs) account for 37% of all deaths, with 275 000 estimated deaths from NCDs (11). Political commitment, sustainable financial mechanism, increased health workforce, ensuring essential NCDs drugs availability at primary healthcare level and community engagement are critical to effectively respond to the NCDs epidemics in Ethiopia (12). However, during the COVID-19 pandemic, the care-seeking behavior of patients with NCDs was severely compromised due to fear, and lack of transportation (13).

According to World Health Assembly in 2020, the Immunization Agenda 2030 (IA2030) strives to reduce morbidity and mortality from vaccine-preventable diseases across the life course (14). Vaccination has been one of the most effective interventions in driving down infant mortality to historically low levels worldwide (15). According to the 2019 Ethiopia Demographic and Health Survey report, the three biggest and most populous regions of Ethiopia (Amhara, Oromia, and SNNPR) constitute 85.5 percent of unimmunized children. The proportion of unimmunized children (for all basic vaccines that are given in the country), per 100 children, is highest in Afar (80%) followed by Somali (64%) and Oromia (60%) (14). Routine vaccination of pregnant women and children must remain a priority during the COVID-19 pandemic response.

The COVID-19 has caused an unprecedented global crisis, including millions of lives lost, public health systems in shock and economic and social disruption, disproportionately affecting the most vulnerable peoples. According to the coronavirus statistics of WHO, globally, as of February, 2023, there are over 675 million confirmed cases and 6.76 million COVID-19 deaths, and in Ethiopia there were about 499 thousand confirmed cases and 7,572 deaths. In Ethiopia, following the confirmation of the first COVID-19 case in the country on 13^th^ March 2020, the national and regional health workforce focused to emergency response mode. Decline in essential health services is attributed to several factors, such as shifting of human resources from essential health services to COVID-19 response, fear of infection due to lack of Personal Protective Equipment (PPE), increased workload due to exposure of health care providers and lack of readiness among health care providers to continue health care services during the pandemic (16). Therefore, this research aimed to assess the status of availability of essential health services during COVID-19 in Ethiopia.

Essential health services are a package of services that improve health outcomes and save lives. Ministry of Health of Ethiopia defined eight areas of essential health services including reproductive, maternal, newborn, child health, and nutrition; major communicable diseases; non-communicable diseases; surgery and injury care; emergency and critical care; neglected tropical diseases; hygiene and environmental health; and health education and behavior change communication (2).

## Materials and Methods

**Study design and setting:** A national mixed-methods study was undertaken. The study was set up in Ethiopia among four regional states and one city administration; namely, Amhara, Oromia, Sidama, Southern Nations, Nationalities, and People's Region (SNNPR), and Dire Dawa City Administration.

**Populations:** The target populations were elements of district healthcare systems, such as district health offices, primary hospitals, health centers, and health posts.

**Sample size and sampling:** In Amhara, Gonder and North Shoa zones were selected. In Oromia West Shewa and Jimma zones were selected. In SNNPR, Hadiya, Gedio, South Omo, Halaba, and Gamo zones were selected. Then, 10, eight, six, and 16 districts, were randomly selected from the selected zones in Amhara, Oromia, Sidama, and SNNPR, respectively. Twenty-eight kebeles (lowest administrative units in Ethiopia) were included from Dire Dawa. A total of 452 health facilities were randomly selected using a lottery method from each selected district resulting in a total of 344 health posts, 92 health centers, and 16 primary hospitals. In Dire Dawa, 15 health centers and 33 health post were included.

Sampling for qualitative study was criteria sampling because people knowledgeable about the issues were included. Four Focus Group Discussions (FDGs) were undertaken; namely with women development army, community leaders, traditional leaders, and hospital quality focal persons. A total of 13 FGDs were undertaken. Pregnant mothers, MCH focal persons in health facilities and health offices, and HEWs were participants of In-depth Interviews (IDIs). A total of 32 people participated in the IDIs.

**Data collection:** Data was collected by face-to-face interview with heads of organizations and facilities, and record review of documents. Moreover, in-depth interviews on barriers of family planning and obstetric services were undertaken and facility observations were done. The tool for data collection was prepared by the World Health Organization. It was tested on district health offices and health facilities which were not part of the cross-sectional survey. The pretest was conducted in Gonder zuriya woreda (Amhara region), Hawassa zuriya woreda (Sidama region), and Harla kebele (Dire Dawa city administration).

The broad areas of the variables that were considered in the study were maternal health service availability (measured on a binary scale); barriers to obstetric care service utilization (measured on a nominal scale); barriers to use of family planning (measured on a nominal scale), sexual, reproductive health and gender-based violence services (measured on a binary scale); and communicable, non-communicable disease prevention, diagnosis, and treatment availability (measured on a binary scale).

Maternal health service in a form of emergency obstetric care was considered available when the six signal functions were performed by the health facility. These are assisted vaginal delivery, removal of retained products and manual removal of placenta, parenteral anticonvulsants, parenteral oxytocics, and parenteral antibiotics.

Antenatal care provision was defined fully and partially on the basis of the examinations and tests expected during a particular visit. Full provision of antenatal care is when all the examinations and tests of a particular visit were done. If at least one examination or test was missing, then it was regarded as a partial provision. Delivery care and neonatal care are defined in a similar fashion.

**Data analysis:** Data was analyzed using SPSS 22.0 software. Descriptive statistical analysis was done. For example, frequency, percentage, and means were calculated. Moreover, basic facilities, such as power source, and water source were compared among regions because we intended to implement region specific interventions. The same was done for availability of Sexual and Reproductive Health diagnostics. The units of analysis were organizations. We did not consider inferential statistics because the aim of the study was to describe the phenomena rather than make causal inference. Moreover, qualitative data was transcribed, translated, and analyzed thematically. The analysis of qualitative data was inductive. Findings were grouped into categories and then into broad themes.

**Ethics:** Ethics clearance was obtained from the Institutional Review Boards of each institution including Dire Dawa University, University of Gonder, Hawassa University, and Jimma University. Participation was voluntary through written consent. The heads of the facilities were informed about the study before they provided the consent to participate. All data were kept confidential.

## Results

The data were collected from 344 health posts, 92 health centers and 16 primary hospitals from Oromia, Amhara, SNNP, Sidama regional states and Dire Dawa City administration (Table 1 provides the distribution of health facilities by region).

**Table 1:** Regional distribution of primary healthcare units, Ethiopia, 2021

Region	Health post, # (%)	Health Center, # (%)	Hospital, # (%)	Total
Oromia	64 (64.7)	32 (32.3)	3 (3.0)	99
Amhara	66 (79.5)	10 (12.1)	7 (8.4)	83
SNNPR	141 (84.9)	22 (13.3)	3 (1.8)	166
Sidama	40 (71.4)	13 (23.2)	3 (5.4)	56
Dire Dawa	33 (68.8)	15 (31.2)	0 (0.0)	48
**Total**	**344 (76.1)**	**92 (20.4)**	**16 (3.5)**	**452**

The findings of this study are organized into seven broad areas. 1) Infrastructure and supplies, 2) maternal and reproductive health service availability during COVID-19, 3) barriers of the utilization of family planning and obstetric services, 4) Availability of communicable disease diagnosis and treatment, 5) Availability of NCD prevention, diagnosis, and treatment, 6) availability and readiness of vaccines, and 7) COVID-19 infection prevention and control.

**Infrastructure and supplies:** Even though 89.6% of health facilities in Dire Dawa had power source, only 43.5% and 34.4% of health facilities in Oromia and Amhara had power source, respectively. Moreover, 45% of health posts had no power source. Functional incinerator was not available in some primary health care units in all regions. Safety boxes were also not fully available (Table 2).

**Table 2:** Percentage of availability of basic facilities during COVID19 by regions, facility type and setting, Ethiopia, 2021

Background characteristics	Basic facilities				
	Power source (%)	Improved water source (%)	Functional incinerator (%)	Telephone service (%)	Safety box (%)
Region					
Amhara	34.4	84.3	33.7	41.7	97.6
Dire-Dawa	89.6	83.3	81.2	88.9	100.0
Oromia	43.5	56.6	42.4	26.7	96.0
Sidama	33.8	66.2	29.3	25.0	94.8
SNNP	24.3	69.1	20.1	20.0	98.2
National average	45.1	71.9	41.3	40.5	97.3
Facility type					
Health Post	45.0	70.4	10.6	NA*	96.7
Health Center	98.7	70.1	93.5	50.6	98.9
Primary Hospital	100.0	100.0	93.8	68.8	100.0
Setting					
Urban	82.2	71.7	66.0	77.1	96.1
Rural	80.7**	37.4	26.0	70.0	97.7

NA* Not assessed **27.4% of rural health facilities reported solar as primary source of electricity

Regarding diagnostic supplies, for example, blood glucose measuring kit was available in 100% of primary hospitals and 80.5% of health centers. The percentage of health facilities with blood glucose measuring kit was the highest in Dire-Dawa (93.8%) and the lowest in Amhara (70.6%). See Table 3.

**Table 3:** Availability of SRH diagnostics and associated items by region, facility type and setting, Ethiopia, 2021

Background characteristics	SRH service diagnostics and associated items							
	Blood glucose %	Dipstick for urine glucose %	Dipstick for urine protein %	Urine test for pregnancy %	Hg test %	HIV test %	Blood cross match %	Mean availability
**Region**								
Amhara	70.6	88.2	82.4	88.2	52.9	94.1	52.9	75.6
Oromia	71.4	82.9	80.0	91.4	68.6	91.4	48.6	76.3
Sidama	87.5	87.5	87.5	100.0	81.3	93.8	56.0	84.8
SNNP	80.0	92.0	92.0	88.0	60.0	88.0	72.0	81.7
Dire-Dawa	93.8	87.5	93.8	93.8	93.8	87.5	68.8	88.4
**Facility type**								
Health Center	80.5	90.8	89.6	96.6	71.2	95.4	64.3	84.0
Primary	100	100	100	100	87.5	100	81.2	95.5
Hospital								
**Setting**								
Urban	85.7	95.2	92.1	95..2	79.3	100	60.3	86.8
Rural	80.0	87.5	90.0	100	65.0	90	67.5	82.9

**Maternal and reproductive health service availability during COVID-19 in Ethiopia:** About 81.4% of woredas and 51.2% of health facilities had defined lists of essential health services before COVID-19. Antenatal care services were fully provided in hospitals and the other services were either partially provided or not available at hospitals (Table 4). About 87.8% of urban health centers did provide family planning services and 72% of rural health centers provided family planning services. Labor and delivery and newborn care services were not fully provided in rural health centers.

**Table 4:** Availability of maternal and child health services at primary hospitals, in Ethiopia, 2021 (n= 16)

Services	Fully n(%)	Partially n(%)	No n(%)
ANC	16(100)	0	0
Labor, delivery and new born care	13(81.3)	3(18.7)	0
Postnatal	15(93.75)	1(6.25)	0
Family planning	13(81.3)	3(18.7)	0
Immunization	14(87.5)	1(6.25)	1(6.25)
Care for sick children	13(81.3)	3(18.7)	0
Diagnosis and treatment of malnutrition	13(81.3)	1(6.25)	2(12.5)

**Figure 1:**
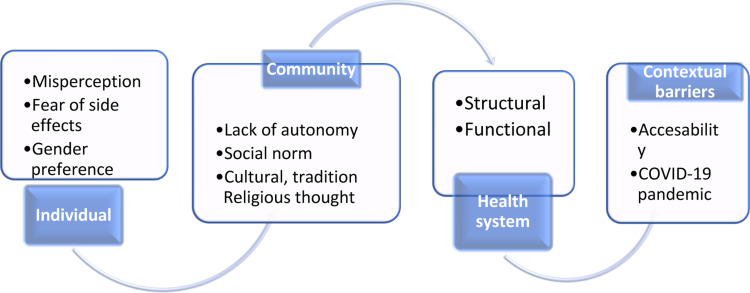
Socio-ecological barriers for family planning service uptake in Ethiopia, 2021

**Barriers of the utilization of family planning and obstetric care service in Ethiopia:** The barriers to use family planning services were thematically summarized under individual barriers, community related barriers, health system related barriers, and contextual barriers (Figure 1). All these barriers were expressed by in-depth interview and focus group discussion participants. According to in-depth interviews and Focus Group Discussions participants, different levels of barriers of obstetric care service utilization were identified. Individual barriers (poor awareness), health system barriers (problems with health system structure and processes), and contextual barriers (internal conflict, cultural and geographical conditions) were identified by the study.

**Availability of communicable- diseases prevention, diagnosis, and treatment**: Communicable disease management, for example, HIV/AIDS diagnosis, prevention and control services, malaria, and tuberculosis were fully provided in 43.5%, 91.3%, and 88% of health centers, respectively.

**Availability of non-communicable disease prevention, diagnosis, and treatment:** Non-communicable disease services, such as cardiovascular disease management was provided by 27.2% of health facilities. Moreover, cervical cancer screening & treatment was provided by 33.7% of health centers. However, only 14% of health centers provided mental health problem diagnosis and treatment. Lastly, 63.1% of health centers were found to provide diagnosis and treatment of diabetes mellitus.

**Availability and readiness of vaccines:** The availability of immunization services was measured with six vaccine antigens and it was found that immunization for children were provided in 84.3% of primary health care units. There was services interruption in about 15% of the primary health care units. Vaccine carrier was available in about 90.7% of selected primary health care units and only 80.1% of primary health care units had full set of vaccine carrier which is a carrier with cold box. The ice pack equipment was fully available in 120 health posts, 82 health centers and 7 primary hospitals. Measles vaccines were available only in 83 health centers and 14 primary hospitals. TT vaccination is not available in all selected health posts. Vaccine readiness was measured with 12 composite indicators which include information on refrigerators, temperature monitoring, and cold chain, among others. At least 60% of the criteria should be met for readiness. The vaccine readiness was 32.4% among the primary healthcare units in the Sidama region while it was only 13.9% in the SNNP region.

**COVID-19 Infection Prevention and Control:** All hospitals and almost all (96.7%) health centers implemented at least one of the following measures to create a COVID-19 safe environment. Nearly two thirds of the hospitals applied COVID-19 infection prevention and control protocols approved by the Ministry of Health, Ethiopia. Nearly 63% of the hospitals and half of health centers designed staff entrance for screening of COVID-19 infection. More than two third of health posts created measures towards COVID-19 safe environment, prepared hand hygiene stations, displayed instructions on hand, prevention and control measures of COVID-19 infections. Nearly halves of health posts were not applying environmental cleaning and disinfection for COVID-19 infection. One fourth of health posts were not using personal protective equipment for their staff. Three-month stock of masks were available only in about 73.8% of PHCUs in Dire-Dawa city at the time of survey and only 44.6% of PHCUs in SNNPR have gown at the time of the survey. More than65% of the hospitals and health centers assigned focal person for COVID-19 service coordination. Nearly sixty nine percent (68.8%) of hospitals and 55.4% health centers have standard operation procedures for the management of patients with suspected or confirmed COVID-19 respectively. Half of the hospitals and half of the health centers performed diagnostic tests for COVID-19 cases. Majority of health centers and primary hospitals had COVID -19 infection prevention and control protocols. Nearly seventy percent of health posts did not have COVID -19 IPC protocols.

## Discussion

About 81.4% of woredas and 51.2% of health facilities had defined lists of essential health services before COVID-19. Antenatal care is provided by nearly 100% of health centers at least fully or partially. However, only 50% of health centers have radiant warmer. Family planning is fully provided 88% urban and 82% rural health facilities.

Only 41% of facilities have functional incinerator. As described in the results section, availability of services for HIV, TB, and Malaria as well as diabetes mellitus are high. Cardiovascular diseases, cervical cancer, and mental healthcare is relatively low. For example, essential non-communicable disease services, such as for cardiovascular disease were provided by only 27.2% of health facilities.

In our study 51.2% of health facilities defined list of essential health services. In WHO regions, 66% of countries defined essential health services to be maintained during COVID-19 (17).

Antenatal care was disrupted in rural areas during COVID-19 in Ethiopia. Bukuluki also reported that sexual and reproductive health services were disrupted in Uganda due to COVID-19 (18).

Only 27.2% of health facilities in Ethiopia provided cardiovascular disease services. In a clinic in South Africa, patients visiting for cardiovascular disease declined by 50% (19). Disruption of follow-up services, reallocation of resources from cardiovascular disease to other services, and distance to health facilities may explain the low level of services provided for cardiovascular disease (20).

Integration of NCD response into future pandemic response plans was recommended to overcome the impact of COVID-19 on NCD services (21).

Like Ethiopia, other countries including Kenya, Mozambique, Uganda, and Zimbabwe also defined antenatal care, delivery care, family planning, and immunization as essential health services before the pandemic (22).

COVID-19 has practical implications on essential health services by disrupting important child, adolescent, maternal, and adult health services. This leads to reversals in gains on improving the situation of children and mothers. Even though, most primary healthcare units defined the essential health services before the COVID-19 pandemic, adequate preparations were not made for infection prevention, HIV care and treatment, and treatment of cardiovascular diseases.

The study is strong in that it considered national sample. Moreover, major providers of primary healthcare, such as health posts, health centers, and primary hospitals were studied. In addition, district health offices as funders and regulators of healthcare services were addresses. However, all the six building blocks of the district health system were not addressed. For example, supply chains for drugs and equipment, and the role of community health volunteers were not studied.

The preparation in defining essential health services among primary healthcare units is high. Moreover, the provision of antenatal care services was high during the pandemic. Nevertheless, neonatal care was suboptimal in half of the health centers. However, infection prevention preparations and the treatment of malnutrition particularly in rural areas were low. Moreover, the provision of HIV care and treatment and chronic diseases was low. Regional health bureaus and partners could work on maintaining maternal health services and make more effort on improving preparations for the provision of services for malnutrition and chronic diseases.
